# Burden of Hypertensive Heart Disease in Iran during 1990–2017: Findings from the Global Burden of Disease study 2017

**DOI:** 10.1371/journal.pone.0257617

**Published:** 2021-09-22

**Authors:** Negar Omidi, Jalal Arabloo, Aziz Rezapour, Farshid Alaeddini, Nicola Luigi Bragazzi, Hamid Pourasghari, Masoud Behzadifar, Mojtaba Salarifar, MohammdRafie Khorgami, Seyyed Mojtaba Ghorashi, Samad Azari

**Affiliations:** 1 Tehran Heart Center, Tehran University of Medical Sciences, Tehran, Iran; 2 Health Management and Economics Research Center, Iran University of Medical Sciences, Tehran, Iran; 3 Laboratory for Industrial and Applied Mathematics (LIAM), Department of mathematics and statistics, York University, Toronto, Canada; 4 Hospital Management Research Center, Iran University of Medical Sciences, Tehran, Iran; 5 Social Determinants of Health Research Center, Lorestan University of Medical Sciences, Khorramabad, Iran; 6 Rajaie Cardiovascular Medical and Research Center, Iran University of Medical Sciences, Tehran, Iran; University of Central Florida, UNITED STATES

## Abstract

**Background:**

Hypertension and its consequent end-organ damage including Hypertensive Heart Disease (HHD) are a major concern that impact health, resulting into impairment and reduced quality of life (QOL). The purpose of this study was to describe the burden of HHD in Iran and comparing it with the World Bank upper middle-income countries (UMICs) in terms of disability-adjusted life years (DALY), mortality and prevalence.

**Methods:**

Using data from the Global Burden of Disease study 2017, we compared the number of DALYs, deaths and prevalence trends for HHD from 1990 to 2017 in all age groups for both sex in Iran, and compared the epidemiology and trends with UMICs and globally.

**Results:**

The age-standardized DALY rate for HHD increased by 51.6% for men (95% uncertainty interval [UI] 305.8 to 436.7 per 100,000) and 4.4% for women (95% UI 429.4 to 448.7 per 100,000) in Iran. The age-standardized prevalence of HHD in Iran was almost twice times higher than globally and 1.5-times more than the World Bank UMICs. The age-standardized death rate for HDD increased by 60.1% (95% UI 17.3 to 27.7% per 100,000) for men and by 21.7% (95% UI 25.85 to 31.48 per 100,000) for women from 1990 to 2017. Age-standardized death rate in Iran was 2.4 and 1.9 times higher than globally and UMICs, respectively.

**Conclusions:**

The higher prevalence and death rate in Iran in comparison with UMICs and globally should encourage health care provider to perform intensive screening activities in at risk population to prevent HHD and mitigate its mortality.

## Background

Hypertension and its consequent end-organ damage including Hypertensive Heart Disease (HHD) are a major concern that impact health, generating impairment and reducing quality of life (QOL) [1, 2]. Current 2017 American Cardiology Association/American Heart Association as well as other international organizations (such as the World Health Organization, WHO) guidelines define hypertension as a systolic blood pressure(SBP) higher than 120 mm hg or a diastolic pressure more than 80mm Hg [3, 4]. HHD with either left or right side heart involvement is associated with increased risk of cardiovascular events [5], systemic vascular resistance, intra-cardiac pressure overload, interstitial fibrosis, concentric left ventricular hypertrophy, diastolic dysfunction, and left atrial enlargement [6]. The spectrum of HHD is highly heterogeneous, spanning from uncontrolled hypertension to either diastolic or systolic heart failure, which has significant mortality and morbidity rates [7].

Recent estimates show that 874 million adults worldwide have a SBP of 140 mm Hg or higher [4]. Prevalence of hypertension tends to increase with aging (79% of men and 85% of women >75 years old have hypertension) [3, 4]. The global burden of hypertension in 2010 was estimated at approximately 1.4 billion and is likely to surpass 1.6 billion by 2025 [8]. In 2016 non-communicable diseases accounted for up to 40.5 million (71%) of global deaths and 17.9 million (44%) of non-communicable deaths were due to cardiovascular disease. Hypertension is the leading risk factor for mortality and disability [9, 10]. In 2010, more than one billion hypertensive adults were living in low- and middle-income countries where the rate of achieving blood pressure goals was 7.7% [8, 10].

The fibrotic remodeling and left ventricular hypertrophy contribute to renin angiotensin aldosterone system activation [11]. According to some global estimates [12]), annual death rate per 100,000 people for SBP of at least 110 to 115 mm Hg increased from 135.6 to145.2 and for SBP of 140 mm Hg or higher increased from 97.9 to 106.3 from 1990 to 2015. Disability-adjusted life-years (DALYs) associated with SBP of at least 110 to 115 mm Hg increased from 148 million to 211 million and for SBP of 140 mm Hg or higher increased from 95.9 million to 143.0 million [12]. To the best of our knowledge, no study has ever been done to comprehensively compare the burden of HHD in Iran and the World Bank Upper Middle-Income Countries (UMICs), stratifying by age and sex incidence and prevalence trends, mortality rates, and compare the overall burden of HHD by country, from 1990 to 2017.

The present study aims at filling in this gap in knowledge. It can provide detailed information to policy- and decision-makers in making informed, evidence-based decisions to prioritize and allocate resources to counteract and mitigate against the burden of HHD. More specifically, these data can be utilized to inform different interventions, from intensive screening aimed at identifying early stages of hypertension-related cardiac damage (HHD), to taking into account trade-offs and opportunities that characterize every screening program. More specifically, the aim of this study is to present a detailed, comprehensive overview of DALY, death and prevalence trends from 1990 to 2017 in all age groups for both men and women in Iran. Moreover, we compare the epidemiology and trends in HHD from 1990 to 2017 in Iran, UMICs and globally.

## Methods

The Global Burden of Diseases, Injuries, and Risk Factors Study 2017 (GBD 2017) estimates incidence, prevalence and mortality rates, years of life lost (YLLs), years lived with disability (YLDs) and disability-adjusted life years (DALYs), for 354 causes by sex, age, and location for 195 countries and territories for 21 regions and 7 super-regions for the years 1990–2017. Detailed description of the GBD 2017 methodology has been provided in previous publications [13–17].

In the current study, we reported numbers and rates of prevalence, death and DALYs of HHD in 2017 and its temporal changes from 1990 to 2017, according to the GBD 2017 data and methodology. These estimates were disaggregated by age group, sex and geographical regions (Iran and the World Bank UMICs group).

The GBD study methods are compliant with the Guidelines for Accurate and Transparent Health Estimates Reporting (GATHER) recommendations [18]. All estimations used in our study are extracted from the GBD 2017 study, the data of which are publicly available online at: http://ghdx.healthdata.org.

### GBD measures and age-standardized estimates

We present data on Iran and the World Bank UMICs group for 1990 and 2017, including prevalence, death and DALYs [13–17].

The GBD 2017 study uses the International Classification of Diseases (ICD)-9 and ICD-10 codes for HHD with *sequelae* (severity levels) where each sequela has a specific disability weight ranging from 0.041 to 0.049. DisMod-MR 2.1, a Bayesian meta-regression tool, was used to estimate non-fatal health losses. This method uses heterogeneous epidemiological data from different data sources to produce internally consistent estimates of incidence and prevalence of HHD [14]. HHD DALYs are the sum of YLDs and YLLs for each location, year, sex and age group. All age-standardized rates were calculated using the GBD world population age standard [16].

### Comparisons with the World Bank UMICs group

The World Bank UMICs were selected for comparison with Iran: they include 56 countries, such as Brazil, the Russian Federation, Indonesia, Iraq and China. UMICs have a Gross national income (GNI) *per capita* between $4,046 and $12,535 [19]. We compared Iran and the above-mentioned group by age-standardized rates of death, and DALYs in 1990 and 2017.

### Uncertainty interval

All data in this study are computed with their 95% uncertainty intervals (UIs) [13–17]. Each parameter was calculated 1,000 times and a distribution with 1,000 samples was produced. The 95% UIs were used to better capture the degree of uncertainty due to the lack of data for some geographic locations (countries/territories) and time periods, and heterogeneity of sources combined together. UIs were derived from the 2.5^th^ and 97.5^th^ values of the 1,000 draws to determine the upper and lower intervals of the 95% UI for each parameter.

## Results

Table 1 shows that there were 276,641(95% UI 235,496–321,324) cases of HHD in 2017 in Iran. Crude prevalence numbers for men and women for all ages increased by 197% and 214.9%, respectively and the age-standardized prevalence rate for HHD increased by 6.2% (95% UI 291.6 to 309.9 per 100,000) for men and 7.2% (95% UI 494.4 to 530.3 per 100,000) for women from 1990 to 2017.

**Table 1:** Prevalence of HHD in men and women in Iran in 1990 and 2017.

**Male**							
	**1990**		**2017**				
**age**	**Number**	**Rate (per 100,000)**	**Number**	**Rate (per 100,000)**	**%μ(Number)**		**%μ(Rate)**
**25–29**	711 (478 to 1,018)	31.3 (21.1 to 44.9)	1,332 (899 to 1,883)	32.6 (22.0 to 46.0)	87.2		4.1
**30–34**	962 (679 to 1,290)	53.1 (37.5 to 71.2)	2,463 (1,732 to 3,318)	55.0 (38.7 to 74.2)	155.7		3.5
**35–39**	812 (542 to 1,123)	57.7 (38.5 to 79.8)	2,377 (1,592 to 3,294)	60.7 (40.6 to 84.1)	192.4		5.1
**40–44**	885 (614 to 1,206)	80.3 (55.78 to 109.4)	2,546 (1,775 to 3,504)	85.5 (59.6 to 117.6)	187.4		6.4
**45–49**	1,526 (1,150 to 1,996)	184.2 (138.8 to 240.8)	5,042 (3,766 to 6,626)	194.7 (145.4 to 255.9)	230.2		5.7
**50–54**	2,555 (1,872 to 3,366)	311.4 (228.2 to 410.2)	6,826 (5,016 to 8,964)	328.7 (241.5 to 431.7)	167.1		5.5
**55–59**	3,603 (2,531 to 4,830)	442.7 (311.0 to 593.6)	8,136 (5,691 to 10,853)	470.1 (328.9 to 627.1)	125.8		6.1
**60–64**	4,639 (3,371 to 6,133)	706.7 (513.6 to 934.4)	10,038 (7,221 to 13,352)	744.9 (535.8 to 990.8)	116.3		5.4
**65–69**	6,246 (4,608 to 8,159)	1,255.1 (926.0 to 1,639.6)	11,459 (8,342 to 15,159)	1,319.1 (960.3 to 1,745.0)	83.4		5
**70–74**	6,096 (4,424 to 8,150)	1,922.0 (1,395.0 to 2,569.7)	12,138 (8,643 to 16,226)	2,032.2 (1447.1 to 2,716.6)	99.1		5.7
**75–79**	2,629 (1,888 to 3,528)	2,716.6 (1,951.0 to 3,645.0)	13,166 (9,394 to 17,586)	2,903.2 (2,071.4 to 3,877.8)	400.6		6.8
**≥80**	3,391.1 (2,599.7 to 4,352.9)	4,461.6 (3,420.3 to 5,726.9)	26,222 (20,200 to 33,836)	4,526.8 (3,487.3 to 5,841.4)	637.2		1.4
**All Ages**	34,357.2 (28,687.3 to 40,495.7)	116.5 (97.3to 137.3)	102,099 (85,783 to 119,426)	244.9 (205.8 to 286.5)	197.1		110.2
**Age-Standardized**	**-**	**291.6 (245.4 to 344.8)**	**-**	309.9 (258.8 to 365.2)	-		6.2
**Female**							
	**1990**		**2017**				
**age**	**Number**	**Rate (per 100,00000)**	**Number**	**Rate (per 100,00000)**	**%μ(Number)**	**%μ(Rate)**	
**25–29**	1,074 (752 to 1,470)	48.2 (33.8 to 66.1)	1,965 (1,375 to 2,720)	49.4 (34.6 to 68.4)	82.9	2.4	
**30–34**	1,406 (1,019 to 1,834)	78.1 (56.6 to 101.9)	3,510 (2,523 to 4,617)	80.1 (57.5 to 105.3)	149.6	2.5	
**35–39**	1,216 (840 to 1,639)	86.7 (59.9 to 116.9)	3,404 (2,348 to 4,599)	89.8 (62.0 to 121.3)	179.8	3.5	
**40–44**	1,355 (966 to 1,793)	124.5 (88.8 to 164.8)	3,761 (2,694 to 4,960)	131.0 (93.8 to 172.7)	177.5	5.2	
**45–49**	2,536 (1,982 to 3,215)	309.7 (242.1 to 392.6)	8,119 (6,402 to 10,208)	328.8 (259.3 to 413.4)	220.1	6.1	
**50–54**	4,128 (3,166 to 5,226)	545.3 (418.2 to 690.3)	11,940 (9,242 to 15,054)	581.1 (449.7 to 732.6)	189.2	6.5	
**55–59**	5,530 (4,139 to 7,087)	789.0 (590.6 to 1,011.3)	14,603.2 (10,934 to 18,775)	844.0 (632.0 to 1,085.2)	164	6.9	
**60–64**	7,006 (5,361 to 8,818)	1,262.4 (966.1 to 1,589.0)	18,502 (14,223 to 23,255)	1,344.9 (1,033.8 to 1,690.4)	164	6.5	
**65–69**	9,245 (7,081 to 11,569)	2,233.8 (1,710.8 to 2,795.3)	22,934 (17,800 to 28,783)	2,384.2 (1,850.5 to 2,992.3)	148	6.7	
**70–74**	8,872 (6,679 to 11,349)	3,368.7 (2,536.0 to 4,609.3)	23,126 (17,415 to 29,675)	3,621.8 (2,727,4 to 4,647.4)	160.6	7.5	
**75–79**	5,033 (3,702 to 6,619)	4,588.9 (3,375.4 to 6,033.8)	22,235 (16,409 to 28,816)	4,979.6 (3,674.9 to 6,453.4)	341.7	8.5	
**≥80**	7,515 (6,005 to 9,438)	7,091.2 (5,666.7 to 8,906.1)	39,871 (31,786 to 50,262)	7,360.8 (5,868.1 to 9,279.1)	430.5	3.8	
**All Ages**	55,422 (47,287 to 63,820)	195.2 (166.6 to 1224.8)	174,541 (148,810 to 201,677)	430.9 (367.4 to 497.9)	214.9	120.7	
**Age-Standardized**	**-**	**494.4 (419.1 to 575.0)**	**-**	**530.3 (452.0 to 617.2)**	**-**	**7.2**	

Table 2 shows that crude death number for men and women for all ages increased by 398.6% and 284.4%, respectively. The most increasing death numbers for men and women are in the octogenarians, respectively (1142% and 553.2%). The age-standardized death rate for HDD increased by 60.1% (95% UI 17.3 to 27.7% per 100,000) for men and by 21.7% (95% UI 25.85 to 31.48 per 100,000) for women.

**Table 2:** Death number and rate of HHD by age and sex in 1990 and 2017 in Iran.

Number of Deaths				Rate of Death (per 100,000) [95% UI]		
age	1990	2017	%μ (1990–2017)	1990	2017	%μ (1990–2017)
**Male**						
**25–29**	5 (3 to 13)	19 (15 to 22)	233.3	0.25 (0.15 to 0.58)	0.47 (0.39 to 0.55)	88
**30–34**	9 (5 to 20)	36 (31 to 42)	284.2	0.52 (0.31 to 1.15)	0.82 (0.7 to 0.94)	57.6
**35–39**	14 (9 to 31)	59 (50 to 69)	304.0	1.05 (0.65 to 2.23)	1.52 (1.30 to 1.78)	44.7
**40–44**	28 (18 to 57)	101 (84 to 117)	253.6	2.61 (5.10 to 5.19)	3.41 (2.83 to 3.96)	30.6
**45–49**	42 (25to 81)	180 (137 to 204)	327.7	5.10 (3.0 to 9.8)	6.97 (5.33to 7.89)	36.6
**50–54**	92 (65 to 167)	309 (242 to 347)	235.1	11.2 (7.9 to 20.4)	14.92 (11.70 to 16.74)	33.2
**55–59**	170 (124.9 to 292.3)	498 (242.9 to 347.5)	193.1	20.9 (15.3 to 35.9)	28.8 (22.27 to 31.73)	37.7
**60–64**	228 (168.6 to 394.2)	633 (489.4 to 696.5)	177.7	34.7 (25.6 to 60.0)	47.0 (36.32 to 51.68)	35.4
**65–69**	275 (203 to 476)	649 (510 to 712)	135.5	55.3 (40.9 to 95.6)	74.7 (58.7 to 81.9)	35
**70–74**	297 (223 to 502)	774 (601 to 855)	160.1	93.9 (70.5 to 158.5)	129.6 (100.6 to 143.1)	38.3
**75–79**	169 (127 to 278)	1079 (850 to 1180)	536.6	175.2 (132.1 to 287.6)	238.0 (187.4 to 260.2)	35.8
**≥80**	315 (246 to 507)	3,921 (2,914 to 4,211)	1142.0	415.4 (323.8 to 667.9)	676.9 (503.0 to 727.1)	62.9
**All Ages**	1,660 (1,246 to 2,818)	8,281(6,387 to 8,852)	398.6	5.63 (4.23to 9.56)	19.8 (15.3 to 21.2)	251.6
**Age-standardized**	**-**	**-**	-	17.3 (13.2 to 28.7)	27.7 (21.2 to 29.6)	60.1
**Female**						
**25–29 years**	13 (8 to 25)	18 (15 to 23)	38.5	0.61 (0.37 to 1.14)	0.47 (0.40 to 0.58)	-22.9
**30–34**	17 (10 to 32)	30 (26 to 37)	77	0.97 (0.60 to 1.83)	0.70 (0.60 to 0.86)	-27.8
**35–39**	22 (14 to 37)	40 (35 to 50)	83.7	1.58 (1.04 to 2.70)	1.07 (0.93 to 1.32)	-32.2
**40–44**	33 (22 to 59)	57 (49 to 71)	71.4	3.09 (2.04 to 5.45)	2.01 (1.74 to 2.48)	-34.9
**45–49**	52 (34 to 90)	104 (92 to 131)	99.6	6.37 (4.21 to 11.03)	4.21 (3.73 to 5.31)	-33.9
**50–54**	94 (63 to 158)	183 (162 to 221)	95.2	12.44 (8.43 to 20.94)	8.94 (7.89 to 10.76)	-28.1
**55–59**	164 (111 to 268)	321 (285 to 384)	95.6	23.48 (15.87 to 38.24)	18.6 (16.5 to 22.24)	-20.7
**60–64**	228 (154 to 374)	517 (430.4 to 610)	126.2	41.22 (27.83 to 67.43)	37.6 (31.29 to 44.37)	-8.7
**65–69**	298 (198 to 490)	662 (558 to 1,369)	121.9	72.09 (47.8 to 118.6)	68.83 (58.01 to 77.55)	-4.5
**70–74**	356 (241 to 578)	882 (703 to 991)	141.5	135.3 (91.8 to 219.8)	138.2 (110.1 to 155.2)	2.1
**75–79**	276 (186 to 447)	1,252 (1009 to 1379)	353.2	251.8 (170.4 to 408.1)	280.4 (226.0 to 308.89)	11.3
≥80	730 (562 to 1109)	4772 (3511 to 5252)	553.2	689.2 (531.0 to 1,046.8)	881.0 (648.2 to 969.6)	27.8
**All Ages**	2,304 (1,638 to 3,645)	8,859 (6,857.5 to 9,969)	284.4	8.12 (5.77 to 12.84)	21.88 (16.9 to 23.94)	169.4
**Age-standardized**	-	-	-	25.85 (18.74 to 40.3)	31.48 (24.21 to 34.41)	21.7
**All Ages (both sexes)**	3,965	17,141	3,321	6.85 (5.00 to 10.89)	20.86 (16.81 to 22.07)	204.5
**Age-standardized (both sexes)**	-	-	-	21.99 (16.44 to 34.29)	29.53 (23.71 to 31.28)	34.2

Tables 3 and 4 shows that the crude number of DALYs for men and women for all ages increased by 272% and 189.9% respectively. Similar to the death numbers, this increase was severe in the octogenarians. Between 1990 and 2017, the age-standardized DALY rate for HHD increased by 51.6% for men (95% UI 305.8 to 436.7 per 100,000 and 4.4% for women (95% UI 429.4 to 448.7 per 100,000).

**Table 3:** DALY number and rate of HHD by age in men in 1990 and 2017 in Iran.

Men						
	1990		2017			
Age	DALY	Rate (per 100,000)	DALY	Rate (per 100,000)	%μ(Number)	%μ(Rate)
**25–29**	410 (259 to 863)	18.1 (11.4 to 38.0)	1278 (1093 to 1495)	31.2 (26.7 to 36.6)	213.6	72.3
**30–34**	613 (394 to 1254)	33.8 (21.7 to 69.2)	2251 (1955 to 2596)	50.3 (43.7 to 58.0)	266.7	48.8
**35–39**	821 (539 to 1662)	58.3 (38.3 to 118.0)	3227 (2771 to 3744)	82.4 (70.7 to 95.6)	292.6	41.3
**40–44**	1395 (926 to 2703)	126.5 (84.0 to 245.2)	4874 (4106 to 5679)	163.7 (137.8 to 190.7)	250.1	29.4
**45–49**	1862 (1154 to 3503)	224.7 (139.3 to 422.7)	7827 (6041 to 8821)	302.3 (233.3 to 340.7)	320.3	34.5
**50–54**	3566 (2583 to 6230)	434.6 (314.8 to 759.2)	11788 (9316 to 13207)	567.7 (448.6 to 636.0)	230.5	30.6
**55–59**	5670 (4253 to 9540)	696.8 (522.7 to 1,172.4)	16388 (12743 to 18069)	947.0 (736.3 to 1,044.1)	188.9	35.9
**60–64**	6531 (4926 to 10980)	995.0 (750.4 to 1,672.8)	17886 (14013 to 19631)	1,327.2 (1,039.8 to 1,456.8)	173.8	33.7
**65–69**	6705 (5064 to 11288)	1,347.5 (1,017.6 to 2,268.5)	15509 (12355 to 16994)	1,785.3 (1,422.2 to 1,956.2)	131.3	32.4
**70–74**	5898 (4541 to 9696)	1,859.6 (1,431.7 to 3,057.2)	15010 (11901 to 16567)	2,513.1 (1,992.6 to 2,773.7)	154.4	35.1
**75–79**	2603 (2002 to 4115)	2,689.0 (2,068.2 to 4,251.7)	16228 (12902 to 17756)	3,578.4 (2,844.9 to 3,915.4)	523.3	33.0
**≥80**	2651 (2104 to 4096)	3,489.0 (2,768.9 to 5,389.0)	33429 (25430 to 36142)	5,771.1 (4,390.2 to 6,239.4)	1160.0	65.4
**All ages**	39,463 (29,644 to 66,465)	133.8 (100.5 to 225.4)	146,966 (117,164 to 158,343)	352.6 (281.1 to 379.9)	272.4	163.5
**Age-Standardized**	**-**	**305.8 (233.5 to 504.6)**	**-**	**436.7 (346.3 to 469.2)**	**-**	**51.6**

**Table 4:** DALY number and rate of HHD by age in women in 1990 and 2017 in Iran.

Women						
	1990		2017			
age	DALY	Rate (per 100,000)	DALY	Rate (per 100,000)	%μ(Number)	%μ(Rate)
**25–29**	917 (595 to 1641)	41.2 (26.7 to 73.8)	1312 (1113 to 1586)	33.0 (28.0 to 39.9)	43.0	-19.9
**30–34**	1093 (721 to 1936)	60.75 (40.0 to 107.5)	2023 (1723 to 2416)	46.1 (39.3 to 55.1)	85.1	-24.1
**35–39**	1230 (848 to 2025)	87.7 (60.4 to 144.4)	2354 (2054 to 2794)	62.1 (54.2 to 73.7)	91.3	-29.1
**40–44**	1658 (1127 to 2808)	152.4 (103.5 to 258.1)	2960 (2595 to 3589)	103.1 (90.4 to 125.0)	78.4	-32.3
**45–49**	2315 (1618 to 3909)	287.2 (197.7 to 477.4)	4950 (4375 to 6011)	200.5 (177.2 to 243.4)	113.8	-30.1
**50–54**	3757 (3663 to 6087)	496.2 (351.7 to 804.0)	7656 (6688 to 9114)	372.6 (325.4 to 443.5)	103.7	-24.9
**55–59**	5648 (3997 to 8860)	805.9 (570.4 to 1,264.2)	11347 (10062.9 to 13143.1)	655.8 (581.6 to 759.6)	108.9	-17.3
**60–64**	6730 (4720 to 10734)	1,212.6 (850.6 to 1,934.2)	15431 (13019 to 17955)	1,121.7 (946.3 to 1,305.1)	129.2	-7.4
**65–69**	7440 (5199 to 11681)	1,797.4 (1,256.3 to 2,822.3)	16,695 (14,397 to 18,820)	1,735.6 (4,496.7 to 1,956.5)	124.3	-3.4
**70–74**	7162 (5040 to 11313)	2,719.4 (1,913.9 to 4,295.3)	17,794 (14,568 to 19,939)	2,786.8 (2,281.5 to 3,122.6)	148.4	2.4
**75–79**	4287 (3017 to 6717)	3,908.5 (2,751.1 to 6,123.3)	19,326 (15,990 to 21,294)	4,328.2 (3,581.1 to 4,769.0)	350.7	13.5
**≥80**	6136 (4835 to 8962)	5,790.3 (4,562.1 to 8,456.5)	40,729 (31,049 to 44,918)	7,519.2 (5,732.0 to 8,292.4)	563.7	29.8
**All Ages**	49,568 (35,145 to 77,129)	174.6 (123.8 to 271.7)	143,699 (120,295 to 157,443)	354.8 (297.0 to 388.7)	189.9	103.2
**Age-Standardized**	-	**429.4 (311.5 to 663.2)**	**-**	**448.7 (370.9 to 490.3)**	**-**	**4.4**

Table 5 compares the indices between Iran, the UMICs and globally. Data shows that the age-standardized prevalence rate (per 100,000) of HHD in Iran has been about two times higher than this rate in the world [420.3 (95% UI 356–489) versus 217.8 (95% UI 184–254)] in 2017. However, the percentage change in this rate in Iran and the world was similar since 1990. Further, the age-standardized death rate (per 100,000) for Iran were 2.5 times higher than the global rate in 2017 [29.53 (95% UI 23.71–31.28) vs 12.28 (95% UI 8.98–13.20)]. The percentage of changes in the age-standardized death rate increased in Iran (34%), while it decreased in the world (-19%). The pattern of the change in DALYs rate is similar to the death rate mentioned above. The age-standardized DALY rate was two times higher in Iran than in the world. Also, between 1990 to 2017, DALYs rate increased in Iran, but it decreased in the world.

**Table 5:** Prevalence, death and DALY rate per 100,000 (age-standardized) for both sexes.

	Prevalence			Death			DALY		
Region	1990	2017	%μ	1990	2017	%μ	1990	2017	%μ
**Iran**	394.0 (332.7 to 459.1)	420.3 (356.0 to 489.7)	6.5	21.99 (16.44 to 34.29)	29.53 (23.71 to 31.28)	34.2	369.99 (277.33 to 575.35)	442.27 (373.04 to 469.65)	19.53
***UMICs***	231.2 (193.2 to 272.5)	266.3 (224.1 to 312.0)	15	23.25 (17.96 to 25.33)	15.54 (10.97 to 16.84)	-33.16	385.19 (292.71 to 418.25)	239.17 (179.56 to 259.26)	-37.9
***Global***	202.8 (170.3to 239.7)	217.8 (184.1 to 254.1)	7.3	15.22 (11.94 to 16.52)	12.28 (8.98 to 13.20)	-19.31	275.59 (218.07 to 298.19)	209.42 (160.54 to 226.28)	-24

Table 6 demonstrated death and DALYs percentages of HHD in all age groups in 1990 and 2017 in Iran, UMICs and globally for all causes. According to the table, the proportion of HHD attributable death to all causes deaths increased four-fold since 1990 accounting for 4.5% of all causes deaths in 2017 in Iran.

**Table 6:** Death and DALYs percentage of HHD in all ages and both sexes in 1990 and 2017 in Iran, UMICs and globally for all causes.

All ages and both sexes	Iran			UMICs			Global		
	1990	2017	%μ	1990	2017	%μ	1990	2017	%μ
**Death [95% UI]**	1.24 (0.9 to 1.97)	4.51 (3.6 to 4.7)	263.7	1.95 (1.46 to 2.12)	2.38 (1.69 to 2.57)	22	1.16 (0.9 to 1.2)	1.65 (1.2 to 1.7)	42.2
**DALYs [95% UI]**	0.39 (0.29 to 0.61)	1.43 (1.2 to 1.62)	266.6	0.76 (0.57 to 0.84)	1.03 (0.78 to 1.15)	35.5	0.43 (0.34 to 0.48)	0.66 (0.5 to 0.74)	53.4

## Discussion

This is the first report evaluating the epidemiology and trends of HHD in Iran and UMICs over the past 27 years.

One of the key findings of the present study is that the number of DALYs has increased from 1990 to 2017 in all age groups for both men and women in Iran. Age-standardized DALY increased in both sexes, more in males than female (51.6% vs. 4. 4%) in Iran. Age-standardized DALY rate was higher in females compared with males (448.7 vs. 436.7) in 2017. The percentage of changes for DALY rate was lower in females during the 27-year period and this could be due to higher baseline DALY rates in 1990. The increase in DALY in both sexes could be attributed to aging, prevalence of hypertension and higher life expectancy [12, 20]. According to the World Bank United Nations Population division data, total life expectancy in Iran increased from 63.8 in 1990 to 75.7 years in 2017. Due to population growth in Iran, 24 million additional people were exposed to HHD in 2017 [20].

The other main finding of the present report was that age-standardized death rate in both sexes increased from 1990 to 2017 in Iran and the increase had a steeper slope in males. The potential cause for higher death rates in females could be explained taking into account the higher prevalence of hypertension and obesity [12, 21]. The increases in death attributed to HHD could be explained by the increase in prevalence of hypertension and improvement in detection of hypertension induced end-organ damage.

The prevalence of HHD increased from 1990 to 2017 in all age groups despite the availability of preventive interventions and potent versatile antihypertensive medications. The higher prevalence in women could be explained by aging (63.8–77) and obesity. These findings support the observation that dietary salt intake, fruit and vegetable consumption, overweight and obesity, and physical activity have also changed substantially over the same time period [12]. Among major cardiovascular risk factors at the global scale, the prevalence of obesity and overweight increased substantially over the last two decades [12]. Overall, about 13% of the world’s adult population (11% of men and 15% of women) and 19.5% of adults in Organization for Economic Cooperation and Development (OECD) countries was obese in 2016, whereas the national prevalence rates of obesity in Iran are 22.7% (15.3% of men and 29.8% of women). Also, there was a significant difference between the prevalence of obesity among males and females in Iran [21–23].

The proportion of HHD attributed mortality to all-cause mortality has increased in Iran from 1990 to 2017. Compared with the UMICs and globally, the proportion of attributed death in Iran was 1.89 and 2.73 times respectively. DALY proportion of HHD to all-cause increased in Iran, UMICs and globally during the aforementioned period, however the proportion of HHD induced disability in Iran in 2017 was 1.38 and 2.16 times higher than UMICs and globally, respectively.

The age-standardized rates demonstrated that the indices in Iran were higher than those of UMICs and globally in 2017. Age-standardized prevalence rate of HHD increased by 15.1% in UMICs. The higher rate of prevalence in Iran compared with UMICs and globally could be explained by higher prevalence of obesity in Iran. The age-standardized prevalence of HHD in Iran was almost twice times higher than globally and 1.5-times more than World Bank UMICs. Age-standardized death rate in Iran was 2.4 and 1.9 times higher than globally and UMICs respectively. Also, age-standardized DALY rate in Iran was 2.11 and 1.8 times higher than globally and UMICs respectively. Comparing results globally and with UMICs during the aforementioned period demonstrated that the age-standardized death and DALY rate per 100,000 decreased from 1990 to 2017 globally and in UMICs, while these trends increased in Iran. This should encourage public health policy-providers to develop and implement robust screening and follow-up program among the general population.

This study and GBD studies used vital registration and administrative hospital data as well as data from representative cross-sectional surveys to inform disease trends across geography and time; these data were adjusted to account for differences in reporting of disease across health systems. Varying practices, resources, patient populations, and quality of data recording, however, likely cause differences in the quality and uniformity of data.

## Conclusions

Age standardized DALY and death rate increased in both sexes from 1990 to 2017 in Iran and this increase was steeper in males. The age-standardized prevalence and death rate of HHD in Iran were almost twice times higher than globally. The higher prevalence and death rates in Iran in comparison with UMICs and globally should encourage public health decision-makers to perform intensive screening activities in at risk population to prevent HHD and mitigate its mortality, specifically, adopting an age and a gender-specific perspective.

## Supporting information

S1 Data (XLSX)

## Abbreviations

HHD: Hypertensive Heart Disease
QOL: Quality of Life
UMICs: Upper middle-income countries
DALY: disability-adjusted life years
GBD: The Global Burden of Diseases
UI: Uncertainty interval
SBP: systolic Blood Pressure
YLLs: Years of life lost
YLDs: Years lived with disability
ICD: International Classification of Diseases
GNI: Gross national income
